# Does environmental regulation truly enhance corporate green environmental investment? Evidence from the supervision of independent directors in listed companies

**DOI:** 10.3389/fpsyg.2024.1430137

**Published:** 2024-09-09

**Authors:** Chao Wang, Feifei Wang, Ping Jiang

**Affiliations:** ^1^School of Management, Hunan Institute of Engineering, Xiangtan, China; ^2^School of Economics, Sichuan University of Science and Engineering, Zigong, China; ^3^School of Business, Hunan University of Science and Technology, Xiangtan, China

**Keywords:** green environmental investment, environmental regulatory policies, independent director supervision, heavy pollution industries, government regulation

## Abstract

With the increasingly prominent environmental issues in China, the government and citizens alike have intensified their focus on corporate investments in green environmental protection. Nevertheless, as government regulations become more stringent, there is substantial debate over whether environmental regulatory policies can consistently encourage listed companies to increase green environmental investments. Simultaneously, independent board supervision plays a crucial role in promoting the compliance and sustainability of listed companies regarding environmental protection. This paper selected a sample of 246 Chinese listed companies from 2010 to 2019, and used a fixed effects model to examine the impact of environmental regulation on the environmental investment of listed companies in China. Moreover, we used a mediation effect model to analyze the role of independent director supervision in influencing the relationship between environmental regulation and companies’ green environmental investment. Additionally, we discuss the heterogeneous impact of environmental regulations on corporate environmental investments. Our findings are as follows: first, during the sample period, the tightening of environmental regulations significantly reduces the growth of environmental investment among the studied firms. As government environmental regulatory policies gradually intensify, the negative impact on environmental investments by listed companies becomes increasingly evident. Second, independent directors help alleviate the adverse impacts of environmental regulations on the environmental investment levels of listed companies. This suggests that the inclusion of independent directors in board governance plays a role in assessing government environmental regulatory policies and overseeing corporate decisions related to environmental investment. Lastly, the heterogeneity analysis indicates that environmental regulation significantly negatively impacts the environmental investment of listed companies in pollution-intensive industries and those located in the western regions. Furthermore, environmental regulatory policies impose greater constraints on the environmental investments of small-sized listed companies compared to their large-sized counterparts.

## 1 Introduction

China’s economy has experienced robust growth and rapid industrialization. However, this economic development has been accompanied by severe environmental pollution, resource depletion, and ecological degradation. These issues profoundly impact the environmental environment and pose significant obstacles to China’s economic transformation and industrial upgrading. As a major global emitter of greenhouse gases, China faces intense pressure from global environmental regulations and extensive international scrutiny.

In response to these grave environmental challenges, the Chinese government enacted the “*Air Pollution Prevention and Control Action Plan*” in September 2013. The strategy explicitly demands strict control over the addition of new capacities in industries with high pollution, as well as the resolute cessation of unauthorized construction projects in industries with severe overcapacity. Simultaneously, China has dramatically emphasized the development of environmental conservation sector in recent years, actively guiding enterprises to invest in the green and environmental sectors. The 2020 China Ecological Environment Statistical Yearbook reveals that investments in controlling environmental pollution amounted to $147.07 billion, representing 1.0% of the GDP. These initiatives underscore the Chinese government’s determination to strengthen environmental governance.

Although the implementation of stringent environmental regulations on high-pollution and energy-consuming industries can significantly contribute to resolving environmental pollution issues and promoting the sustainable and healthy development of the economy in the long term, it may have a particularly adverse impact on China’s economic growth in the short term. Listed companies, as an essential component of China’s economy, with their operational scale and profit-seeking nature, are significant sources of resource consumption and pollution emissions. The government’s strict environmental regulatory policies have stimulated many listed companies to actively develop more environmentally friendly technologies, improve production processes, and procure pollution control equipment. However, due to the uncertainty and long-term nature of the returns on green and environmental investments, and the fact that the development of environmental technologies can occupy significant productive capital, listed companies’ green and environmental investments are not always economically viable. As a result, listed companies demonstrate a diminished willingness and motivation for environmental conservation.

As an important means for the board of directors to govern listed enterprises, independent directors are not involved in the company’s daily operations. They can assess the necessity and effectiveness of environmental investments from a more objective and impartial perspective. Consequently, they are more likely to prioritize the company’s long-term development over short-term profits, supporting long-term green and environmental investments by listed companies. Although these investments may not generate significant financial returns in the short term, they benefit the company’s sustainable development in the long run. Nevertheless, governmental policies on environmental regulations could lead to higher operating expenses for businesses, potentially putting them at a loss in the market of competition. To meet government environmental regulatory requirements, companies must innovate technologically, develop cleaner production processes, and invest in pollution control equipment. These measures may negatively impact the companies’ short-term financial performance, thereby significantly affecting the green environmental investments of listed companies.

As the Chinese government increasingly emphasizes environmental protection and pollution management, raising carbon peak and neutrality to a national priority level, economic development has transitioned from the previous extensive economic growth model to a green and low-carbon development approach. In this context, several questions arise: Will strengthening environmental regulations lead to decreased green environmental investments by listed companies? How can independent directors influence the relationship between environmental regulation and green environmental investment of listed companies? What role do independent directors play in board governance regarding the relationship between environmental regulations and green environmental investment by listed companies? Tackling these issues will enlighten companies about the link between environmental laws and their green investment decisions, and also serve as a roadmap for the government in making environmentally-friendly regulations. Furthermore, it will contribute to a deeper analysis of the influence of independent directors on the relationship between environmental regulations and green investments of listed companies. Thus, it offers empirical evidence to encourage independent directors to actively play a key role in supervising environmental investments and strengthening corporate responsibility for environmental protection.

Under increasingly strict environmental laws and regulations, environmental investment is crucial for achieving green transformation goals. Scholars have focused on three aspects: Porter’s hypothesis on environmental regulation, the impact of environmental regulation on corporate green investment, and the interplay between environmental investment and corporate financial and environmental performance. Porter’s hypothesis on environmental regulation is divided into weak and strong versions. The weak version posits that environmental regulation can promote corporate technological innovation, particularly incentivizing the development of new production processes and clean production technologies (Porter 1991; Porter and Linde, 1995). The strong version suggests that productivity improvements from technological innovation can offset some or all of the additional input costs incurred by the company (Wang et al., 2019; Li et al., 2021; Razzaq et al., 2021).

The question of whether environmental regulations can induce a green investment effect in companies remains controversial. Some scholars argue that command-and-control environmental regulatory policies have promoted green investment in heavy pollution industries (Liu et al., 2023). Others have pointed out that public demands encourage local governments to enforce more stringent environmental regulations, which in turn motivate companies to increase green investments (Liao, 2018; Huang and Lei, 2020; Li et al., 2023). However, some academics have discovered a reverse U-shaped correlation between public participation in environmental regulations and corporate green investment (Li et al., 2023).

Another group of scholars has focused on the relationship between green environmental investments and corporate financial performance and environmental performance (de Burgos-Jiménez et al., 2013; Wang and Zhang, 2020). They discovered a positive correlation between the relative scale of green investments in a nation’s economy and a company’s environmental performance. Simultaneously, strict environmental policies have weakened the positive relationship between green investments and corporate environmental performance, while robust shareholder protection policies have strengthened this relationship (Yan et al., 2021). In addition, some scholars believe that green investments help reduce environmental violations and enhance environmental performance. Moreover, environmental performance can strengthen the impact of green investments on a company’s long-term financial performance (Chen and Ma, 2021). Contrary to these findings, Lee and Min (2015) argue that there is a negative correlation between green *R&D* investments and environmental performance, while green *R&D* investments are positively correlated with financial performance at the corporate level (Lee and Min, 2015).

To sum up, current scholarly works have conducted extensive theoretical and empirical research on the connection between environmental regulation and corporate environmental investments. However, there are still several shortcomings in the existing literature:

First, the current literature on Porter’s hypothesis regarding environmental regulation primarily focuses on how environmental regulations stimulate corporate technological innovation and enhance productivity. It emphasizes whether the benefits of this stimulation can offset the additional input costs caused by environmental regulation. However, it does not analyze how environmental regulation induces corporate environmental investments. Thus, the research results obtained cannot effectively guide corporate decision-making in environmental investments.

Second, existing studies studying the effects of environmental regulations on environmental investment mainly focus on a national or industry level, concentrating on the impact of various environmental regulatory policies on corporate environmental investment. There is limited analysis from a micro-enterprise perspective on the correlation between environmental regulations and green environmental investments in listed companies. Environmental investment and green environmental investment are two different concepts. The concept of environmental investment, which aims to reduce a company’s environmental footprint and decreasing reliance on natural resources to achieve sustainable development, is distinct from green environmental investment. The green environmental investment is more focused on the measures that companies take to reduce their negative impact on the environment, ensuring compliance with government environmental regulations. Therefore, research conclusions and policy recommendations may not be directly applicable to corporate green environmental investment decisions.

Third, the existing literature mainly studies the impact of green investments on corporate environmental performance in a particular country or region and the subsequent effect on corporate financial performance. However, it rarely considers the mechanisms by which government environmental regulatory policies influence corporate green environmental investments. Furthermore, the role of independent director supervision, a potential influencing factor in environmental investment decisions, is not factored into these studies, leading to a lack of understanding of the mechanisms impacting corporate green environmental investments.

Given the shortcomings of the current research and the significance of the research questions, this paper first focuses its research subject at the micro-level of listed companies. The study examines how environmental regulations affect the green environmental investment behavior of listed companies, aiming to explore how environmental regulatory policies influence the decision-making in green environmental investments by listed companies. This study provides new evidence for the government better understand the actual the actual factors that influence corporate green environmental investment decisions. Secondly, this paper introduces the supervision of independent directors to study the link between environmental regulation and corporate green environmental investments. By analyzing the role of independent directors in the mechanism between environmental regulations and green environmental investments by listed companies, this study helps to comprehensively understand how the supervision by independent directors influences the mechanism by which environmental regulatory policies affect corporate green environmental investment behavior. This investigation provides feasible policy recommendations for improving the supervisory behavior of independent directors in listed companies.

The contributions of this paper are aspects. First, by using the supervision of independent directors in board governance as an entry point, it reveals how environmental regulation influences green environmental investments of listed companies, thereby expanding the scope of studies on the factors affecting corporate green environmental investments. Existing literature mainly focuses on the impact of different environmental regulatory policies on corporate green investments and pays less attention to the mechanism of how government environmental regulatory policies affect corporate green environmental investments, including the potential influence of independent director supervision on green environmental investment decisions, which has not been fully considered. Therefore, the study initiates from the supervision of independent directors to explore how environmental regulation affects green environment investments by listed companies. This approach broadens research perspective on the factors affecting corporate green environmental investments and enhances the analysis of the economic impacts of independent director supervision.

Second, this paper enriches research on the factors influencing corporate environmental investment behavior by analyzing green environmental investments from a corporate perspective. Existing studies mainly focus on investigating the impact of environmental regulation on corporate technological innovation, financial performance, and environmental performance from a national or industry perspective. Perspective. However, they pay less attention to the heterogeneity and marginal effects of environmental regulation on corporate green environmental investment. Therefore, this study focuses on the micro-level of listed companies, exploring the heterogeneous effects of environmental regulatory policies on listed companies’ green environmental investments, and further investigates the marginal effects of environmental policies on listed companies’ green environmental investments. This will broaden the scope of studies exploring the link between environmental regulation and green environmental investment behavior, enriching the study of factors influencing corporate environmental investment.

Third, this paper analyzes the impact of green investment of listed companies in different polluting industries, asset size and regions, so as to reduce the risk of green investment for enterprises, alleviate the pressure of their green environmental investments, avoid the decline of green investment of listed companies due to strict environmental policies. This provides practical and empirical evidence for incentivizing enterprises to fulfill their environmental protection responsibilities.

The remainder of the paper is organized as follows: Section 2 reviews the related literature, Section 3 introduces the research methods, Section 4 presents the analysis of empirical results, Section 5 conducts heterogeneity analysis and robustness tests, Section 6 further discusses the marginal effect of environmental regulation on environmental investments in listed companies, and Section 7 summarizes the paper and provides several policy recommendations.

## 2 Literature review and research hypothesis

### 2.1 Research on Porter’s hypothesis

As environmental pollution in China has worsened in recent years, strengthening environmental pollution control has become an essential strategy for the government. Environmental regulation policies, as critical tools for government authorities to manage local environments, have attracted considerable attention from scholars, particularly regarding Porter’s hypothesis (e.g., Jaffe and Palmer, 1997; Wang et al., 2019). For example, Porter and Linde (1995) find that appropriate environmental regulations increase business compliance costs. However, these costs are offset by the compensatory effects of innovation, which further enhance corporate productivity. In contrast, Jaffe et al. (1995) challenge the traditional Porter hypothesis. They argue that while strict environmental regulations promote technological innovation in firms, the cost of such innovation is offset by the internalization of external costs. This improves overall social and environmental welfare but comes at the expense of increasing production costs and reducing corporate competitiveness. Jaffe et al. (1995) study the determinants of environmental innovation in U.S. manufacturing and conclude that environmental regulations do not stimulate technological innovation or enhance the competitiveness of U.S. companies. Moreover, Brunnermeier and Cohen (2003) utilize panel data from the U.S. manufacturing sector from 1983 to 1992 and analyze the impact of environmental regulations on technological innovation. They believe that the increase in environmental enforcement activities does not stimulate corporate innovation. Instead, they suggest that environmental innovation might occur in industries with international competitiveness.

Furthermore, Van Leeuwen and Mohnen (2017) investigate how environmental regulations influence the adoption of green technological innovations by Dutch energy companies and find that environmental regulation promotes green technological innovations in energy companies. However, these innovations do not improve the total factor productivity of the companies, confirming the existence of the weak Porter hypothesis. In contrast to these findings, Gray and Shadbegian (1998) discover that paper mills in states with stringent environmental regulations are more likely to adopt clean production technologies. However, this increases the firms’ investment costs in emissions reduction, leading to a notable decrease in productive investment expenditures in factories with high emission reduction investments, adversely affecting corporate production efficiency. Moreover, as environmental regulatory policies become stricter, environmental regulation has a detrimental impact on green productivity because the innovation compensation effect of environmental regulation is insufficient to offset the environmental compliance costs of firms, indicating the existence of a compliance cost hypothesis (Wang et al., 2019).

To sum up, academics have primarily concentrated on how environmental regulations influence corporate technological innovation and production efficiency. However, the effect of environmental regulations on green environmental investments by Chinese listed companies has not received sufficient attention. In fact, environmental regulations have significant negative impact on the green environmental investment of listed enterprises, because the environmental regulation will crowd out the productive investment funds of enterprises in a short period of time, thus causing adverse effects on the increase of green environmental investment of enterprises.

**Figure 1:**
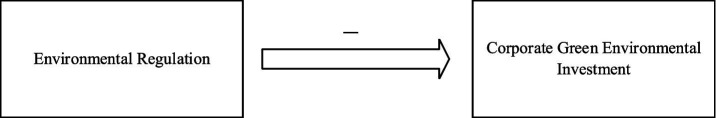
Framework diagram of research hypotheses.

Therefore, to comprehensively analyze the factors affecting green environmental investment of listed enterprises in China, this paper proposes the following research hypotheses (see Figure 1):

*H1*: Environmental regulation negatively affects the increase of corporate green environmental investment.

### 2.2 Research on the relationship between green investment and corporate environmental performance

As global attention to environmental issues, an increasing number of researchers have begun to focus on how corporate green investments impact their environmental performance (Heinkel et al., 2001; Wang et al., 2014). For example, Li and Ramanathan (2020) find that in regions with better institutional environments or higher *FDI*, increasing green investments can lead to more significant improvements in environmental performance. They categorize green investments into pollution control investments and pollution prevention investments, noting that pollution prevention investments have a more substantial positive impact on environmental performance, while pollution control investments do not significantly affect environmental performance. In contrast to these findings, Indriastuti and Chariri (2021) use data from 132 manufacturing companies listed on the Indonesia Stock Exchange to study the relationship between green investments, corporate social responsibility (*CSR*), and sustainable performance. Their research reveals that green investments positively impact a company’s sustainable performance.

In addition, some scholars have shifted their research focus toward corporate financial performance. For example, Schaltenbrand et al. (2018) discover that an employer’s financial performance significantly influences managers to increase the company’s green investments. Differing from this research perspective, Nakamura (2011) uses 3,237 Japanese companies to study the impact of environmental investments on corporate financial performance, finding that environmental investment had no significant effect on corporate financial performance in the short term. However, environmental investments significantly improve a company’s financial performance in the long term, although the positive effect of these environmental investments on the company’s financial performance disappears in the following period.

Moreover, due to the significant relevance of the link between CSR and environmental performance for corporate executives and governments, this area has received much attention from researchers in recent years. For instance, Chuang and Huang (2018) believe that CSR significantly positively impacted green technology investment, promoting improved corporate environmental performance. Contrary to these findings, Shaukat et al. (2016) argue that corporate stakeholders need to strengthen the CSR orientation within the board of directors when evaluating the company’s CSR activities because they believe that the stronger the CSR positioning of the board of directors, the higher its environmental and social performance.

To sum up, current studies mainly concentrate on how green investments effect corporate environmental performance in specific countries or regions and verify the effect of corporate environmental performance on improving financial performance. Additionally, some scholars have recognized the influence of diverse characteristics, such as board gender, on corporate environmental performance. However, the potential influence of independent director supervision on environmental investment decisions has not been adequately considered. This results in a lack of in-depth research on the indirect impact of independent directors on corporate environmental investment. Thus, it fails to analyze how independent directors influence corporate compliance with government environmental regulations and corporate environmental investment behavior. Therefore, this paper focus on independent director and analyzes how environmental regulatory policies affect corporate green environmental investments by influencing the decisions of independent directors.

**Figure 2:**
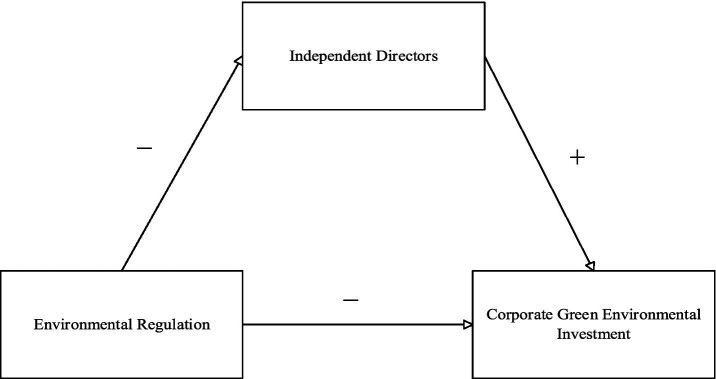
Framework diagram of research hypotheses.

Based on the above analysis, this paper suggests that environmental regulation may have an impact on corporate green environmental investment decisions through the mediating variable of independent director, and proposes the following research hypothesis (see Figure 2):

*H2*: Independent director supervision plays a mediating role between environmental regulation and corporate green environmental investment.

### 2.3 Research on the link between environmental regulation and corporate green investment

At present, environmental supervision has become the main driving force for attracting green investments from enterprises and realizing sustainable green economic development. Consequently, scholars have shown considerable interest in studying the connection between environmental supervision and green investments of enterprises (Chang and Wang, 2010; Gu et al., 2021; Li et al., 2021). Some scholars argue that environmental regulations can be a crucial driving factor for inducing corporate green investment. When governments implement stricter environmental regulations, companies usually face higher compliance costs, motivating them to adopt environmental measures to meet regulatory requirements (Xu et al., 2022). For instance, Ren et al. (2022) suggest that improving energy conservation and emission reduction efficiency and enhancing technological innovation capabilities can enhance the positive impact of green investments on low-carbon emission reduction.

In fact, the influence of different environmental regulations on corporate green investments varies greatly. For instance, Liu et al. (2023) find that the implementation of the “*New Environmental Law*” significantly promotes green investments in heavily polluting industries. This promotional effect is primarily due to the law leading to more standardized corporate environmental information disclosure, stricter environmental enforcement, and tighter financial constraints. Consequently, it encourages companies to make green investments by companies. In contrary, Eyraud et al. (2013) study the determinants of green investments in 35 developed and emerging countries. They find that introducing carbon pricing mechanisms and policy interventions requiring green energy positively impacted green investments. However, biofuel support measures do not noticeably impact corporate green investments.

The above studies demonstrate that scholars have rich findings on the relationship between environmental regulations and firms’ green investment behavior, which provide valuable guidance for studying the effect mechanism of environmental regulation on green environmental investment. However, the existing literature mainly focuses on the impact of different types of environmental regulatory policies on corporate environmental investments and less on analyzing heterogeneous relationship between government environmental regulatory policies and corporate green environmental investment green environmental investments by listed companies from a micro-enterprise perspective. In fact, corporate green environmental investment and green environmental investment are two completely different concepts. The goal of corporate environmental investment is to reduce the company’s environmental footprint, reduce dependence on natural resources, and thus achieve sustainable development of the enterprise. In contrast, corporate green environmental investments are more focused on companies taking steps to minimize their negative impact on the environment and encourage companies to meet government environmental regulations. Therefore, the research conclusions and policy recommendations derived from the impact of environmental regulation on the heterogeneity of environmental investment are not applicable to green environmental investment decisions at the enterprise level.

**Figure 3:**
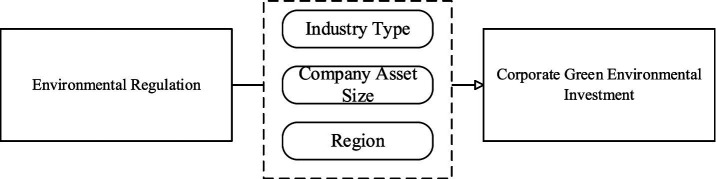
Framework diagram of research hypotheses.

Based on the above analysis, this paper focuses on the green investment of enterprises and investigates the heterogeneous impact of environmental regulation on the green investment of Chinese enterprises, and propose the following research hypotheses (see Figure 3):

*H3-1*: There are significant differences in the impact of environmental regulations on green environmental investment of listed enterprises in different polluting industries.

*H3-2*: There are significant differences in the impact of environmental regulations on green environmental investment of listed companies with different asset sizes.

*H3-3*: There are significant differences in the impact of environmental regulations on green environmental investment of listed companies in different regions.

## 3 Research design

### 3.1 Data descriptions

In order to analyze the impact mechanism of environmental regulation on listed firms’ green environmental investment, this paper selects the panel data of Chinese A-share listed firms from 2010 to 2019 as the research sample, and the reason for selecting 2010–2019 as the sample period is that there is a data lag and missing data problem in the publication of environmental and economic statistics. Meanwhile, in view of the availability of data, to ensure the validity of data and eliminate the influence of abnormal values on regression results, the following treatments were applied to research samples: (1) Exclusion of samples from companies in the financial sector, as well as ST, *ST, and PT companies; (2) Exclusion of listed companies that have issued both A-shares and B-shares; (3) Exclusion of samples that did not disclose the amount of environmental investments and other samples with missing related data; (4) To reduce the influence of outliers on the research conclusions, all continuous variables were winsorized at the 1st and 99th percentiles. Two thousand four hundred and sixty observations from 246 listed companies were obtained after excluding samples that did not disclose environmental investment amounts and missing data.

Regarding data sources, the data on environmental investments was obtained from the annual CSR reports published by the companies. These reports are mandated by the Shenzhen Stock Exchange’s “*Guidelines for Social Responsibility of Listed Companies*” (2006) and the Shanghai Stock Exchange’s “*Guidelines for Environmental Information Disclosure of Listed Companies*” (2008), ensuring the availability of data related to environmental investments. The data on the intensity of environmental regulation were sourced from the CSMAR database and the China Economic Information Network statistical database (CEIdata), while data on independent directors came from the CSMAR. Other company-level variable data were obtained from the Wind database.

### 3.2 Research methods

#### 3.2.1 Benchmark regression model

To explore the impact of environmental regulation policies on green environmental investments of listed companies and further analyze the mechanism by which environmental regulation policies influence the level of green environmental investments through political connections, this paper, based on Porter’s Hypothesis theory (Porter and Linde, 1995), constructs the following panel data econometric model:


(1) $$\mathrm{EnvirExpen}d_{i,t}=\beta_{0}+\beta_{1}ER_{i,t}+\delta \sum \mathrm{Contro}l_{i,t}+\mu_{i}+\varepsilon_{i,t}$$

Where $ER_{i,t}$ represents the intensity of environmental regulation; $\mathrm{EnvirExpen}d_{i,t}$ represents the level of green environmental investment by listed companies; $\sum \mathrm{Contro}l_{i,t}$ represents control variables that influence environmental investment, including the nature of state-owned enterprises (*SOE*), company establishment years (*FirmAge*), company asset size (*Size*), return on equity (*ROE*), shareholding percentage of the largest shareholder (*Top*), asset-liability ratio (*Lev*), revenue growth rate (*Growth*), and cash flow ratio (*Cashflow*). $\mu_{i}$ represent year and company fixed effects, and $\varepsilon_{i,t}$ are random disturbance terms.

#### 3.2.2 Mediation effect model

In order to study the role of environmental regulation through independent directors to influence the role of green environmental investment in listed enterprises,

referring on the stepwise method proposed by Judd and Kenny (1981) and Baron and Kenny (1986), this paper introduces Equation 2 and Equation 3 to construct the mediation effect model. Equation 2 examines the influence of environmental regulations on independent directors of listed companies. Equation 3 introduces independent directors of listed companies from Equation 1 to test mediation effect of independent directors of listed companies between environmental regulation and green environmental investment. The research model is outlined as follows:


(2) $$I{\mathrm{ndep}}_{i,t}=\beta_{0}+\alpha_{1}ER_{i,t}+\delta \sum \mathrm{Contro}l_{i,t}+\mu_{i}+\varepsilon_{i,t}$$

(3) $$\begin{array}{l} \mathrm{EnvirExpen}d_{i,t}=\eta_{0}+\eta_{1}ER_{i,t}+\eta_{2}\mathrm{Inde}p_{i,t}\\ \;+\delta \sum \mathrm{Contro}l_{i,t}+\mu_{i}+\varepsilon_{i,t} \end{array}$$

Where $ER_{i,t}$ represents the intensity of environmental regulations; $\mathrm{EnvirExpen}d_{i,t}$ represents the level of green environmental investment by listed companies; $I{\mathrm{ndep}}_{i,t}$ represents the proportion of independent directors. $\sum \mathrm{Contro}l_{i,t}$ represents other control variables that affect green environmental investment. $\mu_{i}$ represent year and company fixed effects, and $\varepsilon_{i,t}$ are random disturbance terms.

### 3.3 Variable definitions

#### 3.3.1 Green environmental investment (*EnvirExpend*)

Green environmental investment helps enhance the company’s social image and reputation, promotes technological innovation and sustainable development, and reduces environmental risks. The environmental investments reported in CSR reports of listed companies mainly include investments in environmental technology research and development, procurement and transformation of environmental protection equipment, pollution control, clean production, environmental management expenditures, and ecological protection. Drawing on the study by Chen and Ma (2021), this paper aggregates these environmental investment indicators and uses the ratio of green environmental investment to operating income to measure the intensity of green environmental investments by listed companies. This ratio serves as the core explanatory variable (*EnvirExpend*) in this study. The use of the relative ratio of green environmental investments aims to mitigate the effect of company size on the regression results.

#### 3.3.2 Environmental regulation (*ER*)

With the continuous increase in environmental protection requirements, companies must comply with stringent environmental laws and standards to meet government regulatory demands and reduce environmental risks. Companies must invest funds and resources to construct environmental protection facilities and innovate technology to reduce environmental pollution and emissions to meet these requirements. Therefore, environmental regulations can impact environmental investment choices and encourage the adoption of more environmentally friendly production methods and equipment. Following the approach by Wang and Zhang (2022), this paper uses the proportion of completed investments in industrial pollution control to the added value of the secondary industry to measure the intensity of environmental regulation.

#### 3.3.3 The supervision of independent directors (*Indep*)

The supervision of independent directors is essential for the governance of listed companies. When major shareholders or actual controllers have dominant control rights, independent directors can protect the interests of small and medium-sized investors. Moreover, a larger proportion of independent directors on the board can help management control potential investment decision risks by utilizing their professional knowledge and experience. Therefore, independent directors improve the efficiency of supervisory decisions in management and governance capacity, promoting the long-term healthy development of the company. Referencing the study by Chintrakarn et al. (2021), this study uses the proportion of independent directors to the total number of board members to represent the supervision of independent directors.

#### 3.3.4 Control variables

Drawing on existing research (Huang and Lei, 2020; Zhao et al., 2022), this paper selects the nature of state-owned enterprises (*SOE*), the age of firm establishment (*FirmAge*), the size of firm assets (*Size*), the firm’s profit after tax divided by net assets (*ROE*), the percentage held by the largest shareholder (*Top*), liabilities to total assets (*Lev*), operating income growth rate (*Growth*), and cash flow ratio (*Cashflow*) as control variables (see Table 1). These variables potentially impact the green environmental investments of listed companies. To control for potential time and individual effects, this paper includes fixed effects for years and companies in the model.

**Table 1:** Control variable definition.

Variable	Name	Calculation method	Data source
*ROE*	Return on equity	Net profit/average shareholders’ equity	CSMAR database
*SOE*	Whether it is a state-owned firm	The value of state-owned firms is 1, while the value of other types is 0	CSMAR database
*Growth*	Revenue growth rate	Current year operating revenue/last year operating revenue −1	CSMAR database
*Lev*	Asset-liability ratio	Total liabilities at the end of the year divided by total assets at the end of the year	CSMAR database
*FirmAge*	The year of establishment	Ln (current year-the year of establishment +1)	CSMAR database
*Cashflow*	Cash flow ratio	Net cash flow from operating activities divided by total assets	CSMAR database
*Size*	Firm size	The natural log of total assets at year end	CSMAR database
Top	Shareholding ratio of the largest shareholder	Number of shares held by the largest shareholder/total number of shares	CSMAR database

## 4 Empirical result analysis

### 4.1 Descriptive statistics and correlation analysis

The descriptive statistics of the variables are presented in Table 2. The maximum and minimum values of *ER* are 0.010 and 0.001, respectively, indicating that the intensity of environmental regulation in listed companies is relatively low. Meanwhile, the median value of green environmental investment (*EnvirExpend*) is less than the mean value of corporate environmental investment. This indicates that the green environmental investment of most listed companies is below the average green environmental investment during the sample period. The mean and median values of the proportion of independent directors (*Indep*) are 0.370 and 0.333, respectively. It shows that the mean value of *Indep* is higher than the median value of *Indep*, indicating that the proportion of independent directors in most listed companies is below the average.

**Table 2:** Descriptive statistical analysis of variables.

Variable	Obs	Mean	SD	Min	Median	Max
EnvExpend	2,460	0.177	0.351	0.000	0.057	2.238
ER	2,460	0.003	0.002	0.001	0.002	0.010
Indep	2,460	0.370	0.048	0.333	0.333	0.533
S0E	2,460	1.498	0.695	1.000	1.000	5.000
FirmAge	2,460	2.836	0.334	1.609	2.890	3.434
Size	2,460	22.489	1.291	19.781	22.414	25.971
ROE	2,460	0.059	0.146	−0.688	0.059	0.506
Top	2,460	0.363	0.150	0.105	0.351	0.789
Lev	2,460	0.493	0.208	0.055	0.503	0.989
Growth	2,460	0.209	0.530	−0.481	0.108	3.541
Cashflow	2,460	0.048	0.072	−0.174	0.047	0.260

Among all control variables, the size of firm assets (*Size*) has the largest standard deviation, with a significant gap between the maximum and minimum values, indicating that the scale of listed companies fluctuates greatly with a polarization phenomenon. Moreover, among other control variables, Cashflow has the lowest standard deviation, and its mean and median are approximately equal, suggesting that the fluctuation of the cash flow ratio (*Cashflow*) of listed companies during the sample years is not significant, presenting a symmetric distribution with a low degree of dispersion.

The correlation results among the variables are shown in Table 3. The correlation coefficient between *EnvirExpend* and *ER* is-0.070, which is significant at the 1% level, indicating a notable negative correlation between green environmental investment and the intensity of environmental regulation. However, the correlation coefficient between *EnvirExpend* and *Indep* is-0.031, and their correlation is statistically insignificant. This indicates that there is no correlation between the green environmental investment of listed companies and the proportion of independent directors. Furthermore, the correlation coefficient between *Indep* and *ER* is also insignificant, indicating that the strengthening of environmental regulation does not significantly influence the supervision of independent directors in listed companies.

**Table 3:** Correlation analysis among variables.

Variable	|EnvExpend	ER	Indep	S0E	FirmAge	Size	ROE	Top	Lev	Growth	Cashflow
EnvExpend	1.000										
ER	−0.070***	1.000									
Indep	−0.031	−0.031	1.000								
S0E	−0.048**	−0.097***	0.030	1.000							
FirmAge	0.012	0.000	0.032	−0.120***	1.000						
Size	−0.269***	0.060***	−0.018	−0.215***	0.023	1.000					
ROE	0.050**	−0.028	−0.035*	0.101***	−0.045**	0.010	1.000				
Top	0.064***	0.109***	0.043**	−0.141***	−0.105***	0.239***	0.027	1.000			
Lev	0.012	0.033	−0.002	−0.240***	0.196***	0.262***	−0.232***	0.165***	1.000		
Growth	0.020	−0.048**	0.002	0.066***	−0.039*	−0.027	0.250***	−0.001	0.026	1.000	
Cashflow	0.057***	0.000	−0.049**	0.030	0.069***	0.082***	0.312***	0.089***	−0.134***	0.019	1.000

***, **, and * Denote the significance at the 1, 5, and 10% levels, respectively.

### 4.2 Baseline regression analysis of the impact of environmental regulation on corporate environmental investment

This study utilized a Hausman test on a panel data model to examine the influence of environmental regulation on corporate environmental investments. The results of the test indicated that the null hypothesis was rejected, implying that a fixed effects model is appropriate for the study. The main findings of baseline regression are presented in Table 4, where Column (1) only considers individual company effects and Column (2) considers both individual company effects and year effects.

**Table 4:** Benchmark regression results of the impact of environmental regulation on firms’ environmental investment.

Variable	(1)	(2)
*ER*	−10.311***	−10.344***
	(−2.88)	(−2.89)
*S0E*	0.028	0.028
	(0.89)	(0.88)
*FirmAge*	−0.149***	−0.124
	(−2.78)	(−0.67)
*Size*	−0.169***	−0.169***
	(−8.02)	(−8.01)
*ROE*	−0.009	−0.010
	(−0.19)	(−0.20)
*Top*	−0.134	−0.133
	(−1.20)	(−1.19)
*Lev*	−0.053	−0.056
	(−0.69)	(−0.75)
*Growth*	0.007	0.007
	(0.49)	(0.49)
*Cashflow*	0.033	0.034
	(0.23)	(0.24)
*Constant*	4.463***	7.720
	(8.89)	(0.35)
*R-squared*	0.206	0.206
*Number of listed companies*	246	246
*Company FE*	YES	YES
*Year FE*	NO	YES

*t*-values are in parentheses, ***, **, and * denote the significance at the 1, 5, and 10% levels, respectively. To prevent possible heteroscedasticity and autocorrelation problems, the firm-level clustered robust standard errors was used to estimate the model.

First, increased environmental regulation (*ER*) has significantly reduced the environmental investment level of listed companies. As seen in Columns (1) and (2) of Table 3, the regression coefficients of *ER* are significantly negative at the 1% level. This suggests that heightened environmental regulations result in decreased environmental investments made by listed companies, meaning that more stringent environmental regulations have a notable impact on limiting the growth of corporate environmental investments. This study’s findings are consistent with those of Hu et al. (2023), who argue that environmental regulations significantly inhibits corporate green environmental investments, mainly because the adoption of green credit policy leads to elevated financing expenses for companies characterized by high levels of energy consumption and pollution, further increases the marginal cost of environmental investment, and ultimately leads to the reduction of enterprises’ enthusiasm for environmental governance. On the one hand, improving the intensity of environmental regulations will increase the additional production costs for enterprises. Enterprises need more funds to buy more expensive environmental protection equipment to meet the environmental protection standards, and these additional costs will squeeze the funds available for environmental protection investment. Furthermore, the uncertainty and long-term nature of the returns on corporate environmental investments make companies reluctant to invest in clean production technologies (Wang and Zhang, 2020). Especially for companies in poor financial condition or with low credit ratings, an increase in the intensity of environmental regulation may further restrict their financing capacity, ultimately leading to a significant negative impact of environmental regulations on corporate environmental investments.

Second, increasing company size (*Size*) significantly hinders firms’ environmental investments. Whether the annual time effect is considered or not, the coefficient of *Size* is significantly negative at the 1% level, suggesting that the growth in firm size of listed companies significantly impedes the environmental investment of listed companies. The potential reason is that larger companies often face greater economic pressures during their operations, such as higher operating costs and more debt burdens. These pressures may make companies more cautious in the face of environmental investments and may even prioritize short-term economic gains over long-term environmental responsibilities.

Finally, when only individual effects are considered, the age of company (*FirmAge*) exerts a notable adverse impact on the growth of environmental investments. Column (1) in Table 4 shows that the coefficient of *FirmAge* is-0.149, which is significant at the 1% level. This suggests that the longer a listed company has been established, the less willing it is to increase environmental investments. This result may be due to increased environmental regulations, which have led to higher barriers to entry in certain industries. The longer establishment of listed companies has maintained relatively advanced technological innovation in their industries. At this point, companies might reduce their environmental investments to lower operating costs and maintain a competitive edge.

### 4.3 Addressing the endogeneity issue between environmental regulation and green environmental investment of listed companies

Since some of the environmental investment information disclosed by the sample listed companies is used to pay pollution fees, there may be an endogeneity issue between environmental regulation and corporate green environmental investments. In order to address the issue of endogeneity between environmental regulation and corporate green environmental investments in research findings, this paper introduces the average intensity of environmental regulation of listed companies in registered provinces (*ER_AVER*) as an instrumental variable in dealing with endogenous problems. The main reason for using *ER_AVER* as an instrumental variable is that the average intensity of environmental regulation for listed companies in registered provinces is calculated as the mean value of the intensity of environmental regulation in the provinces where the listed companies are located. Thus, *ER_AVER* is highly correlated with the intensity of environmental regulation at the government level. Furthermore, the provinces’ average intensity of environmental regulation over different periods is exogenous to the micro-level data of the listed companies’ environmental investments. The instrumental variable for the average environmental investment of listed companies meets the exogenous requirements. Therefore, this study selects the mean value of environmental investments in the provinces where the listed companies are incorporated as an instrumental variable.

By employing the *2SLS* method for estimation, the regression results are shown in Table 5. The instrumental variable relevance test indicates that the *p*-value of the Kleibergen-Paap rk LM statistic is below 0.1, indicating that the null hypothesis of insufficient identification of instrument variables can be rejected. The Cragg-Donald Wald F statistic exceeds the critical value of 8.96 for Stock-Yogo’s 15% bias, leading to the rejection of the null hypothesis regarding the existence of weak instrumental variables. Thus, the choice of instrumental variables is deemed suitable.

**Table 5:** Treatment of endogeneity issues.

	(1)	(2)
	IV-2SLS	IV-2SLS
*ER*	−16.085***	0.222
	(−2.63)	(0.02)
*S0E*	0.027	0.028
	(1.11)	(1.02)
*FirmAge*	−0.146***	0.050
	(−3.69)	(0.45)
*Size*	−0.169***	−0.177***
	(−14.36)	(−14.68)
*ROE*	−0.012	−0.048
	(−0.32)	(−1.22)
*Top*	−0.124	−0.060
	(−1.36)	(−0.66)
*Lev*	−0.050	−0.085
	(−0.96)	(−1.62)
*Growth*	0.007	0.003
	(0.50)	(0.21)
*Cashflow*	0.033	0.017
	(0.31)	(0.16)
*Constant*	4.476***	4.258***
	(14.78)	(10.59)
*Number of listed companies*	246	246
*Company FE*	YES	YES
*Year FE*	NO	YES
*Kleibergen-Paap rk LM statistic*	294.05	111.12
	[0.000]	[0.000]
*Cragg-Donald Wald F statistic*	766.50	269.48
*Stock-Yogo 15% critical values*	8.96	8.96

The *z*-statistics are in round brackets and the p-values are in center brackets. ***, **, and * Denote the significance at the 1, 5, and 10% levels, respectively. To control for possible heteroskedasticity and autocorrelation, firm-level clustering robust standard errors are used for SYS-GMM, while industry-level clustering robust standard errors are used for 2SLS.

Analyzing the regression results obtained by the 2SLS method in Table 5, we find that when time effects are not considered, the results in Column (1) show that the coefficient of *ER* is-16.085 and significant at the 1% level, indicating that the increase in the intensity of environmental regulation has hindered the increase in environmental investments by listed companies. This conclusion is consistent with the baseline regression results. However, when considering both individual company effects and time effects are considered, the results in Column (2) show that the coefficient of *ER* becomes insignificant, indicating that the increase in the intensity of environmental regulation does not significantly affect the environmental investments of listed companies. Upon examining the endogenous problem regarding environmental regulation and corporate environmental investment, the influence of environmental regulations on the environmental investment of listed companies is uncertain. In order to better understand how environmental regulations affect environmental investments, it is crucial to investigate the impact mechanism of environmental regulations on the environmental investments of listed companies.

### 4.4 Analysis of the mechanism influence of independent directors on the relationship between environmental regulation policies and environmental investment of listed companies

From the previous analysis in Table 4, we find that the tightening of environmental regulations greatly impedes the growth of green environmental investments made by listed companies. However, there seems to be a lack of consistency in the influence of environmental regulation on the green environmental investments of listed companies when accounting for the endogeneity between the two factors.

Independent directors’ attitude toward environmental protection in board governance plays an important role in the development and implementation of corporate sustainability programs (Cosma et al., 2021), and they are typically not controlled by the internal management or major shareholders, enabling them to more objectively assess government environmental regulation policies and monitor corporate green environmental investment decisions. Consequently, companies with more independent directors are likely to invest more in the environment (Bhuiyan et al., 2021). It can be seen that environmental regulatory policies can influence firms’ green environmental investment decisions by influencing the decisions of independent directors (Farza et al., 2022). Thus, environmental regulation may have an impact on corporates’ green environmental investment decisions through the mediating variable of independent directors. Therefore, this paper attempts to introduce the variable of the proportion of independent directors (Indep) and uses the stepwise testing method (Judd and Kenny, 1981; Baron and Kenny, 1986) to explore the mechanism by which independent directors influence the impact of environmental regulation on the green environmental investments of listed companies. The research results are presented in Table 6.

**Table 6:** Mediation effect test results.

Variable	Model (1)	Model (2)	Model (3)
	EnvirExpend	Indep	EnvirExpend
*ER*	−10.344***	−1.046**	−10.718***
	(−2.89)	(−2.25)	(−3.03)
*Indep*			−0.358**
			(−2.13)
*S0E*	0.028	0.008	0.031
	(0.88)	(1.58)	(0.97)
*FirmAge*	−0.124	0.020	−0.117
	(−0.67)	(0.74)	(−0.64)
*Size*	−0.169***	−0.000	−0.169***
	(−8.01)	(−0.01)	(−8.03)
*ROE*	−0.010	−0.013	−0.014
	(−0.20)	(−1.60)	(−0.29)
*Top*	−0.133	−0.006	−0.135
	(−1.19)	(−0.46)	(−1.22)
*Lev*	−0.056	−0.007	−0.059
	(−0.75)	(−0.82)	(−0.78)
*Growth*	0.007	−0.001	0.007
	(0.49)	(−0.60)	(0.47)
*Cashflow*	0.034	0.018	0.040
	(0.24)	(1.22)	(0.28)
*Constant*	7.720	2.201	8.508
	(0.35)	(0.71)	(0.39)
*R-squared*	0.206	0.012	0.208
*Number of listed companies*	246	246	246
*Company FE*	YES	YES	YES
*Year FE*	YES	YES	YES

The values of *t* are in parentheses, ***, **, and * denote the significance at the 1, 5, and 10% levels, respectively. To control for possible heteroskedasticity and autocorrelation, firm-level clustering robust standard errors were used for the model.

The research results show that the coefficient of *ER* is significantly negative at the 1% level in Column (1) of Table 6, indicating that an increase in the intensity of environmental regulation significantly hinders the improvement of environmental investment levels of listed companies. At this point, the regression coefficient (c) for ER stands at-10.344, indicating that the total effect of environmental regulation (*ER*) on the environmental investment (*EnvirExpend*) of listed companies is-10.344. As shown in Column (2) of Table 6, when considering the impact of environmental regulation (*ER*) on independent directors (*Indep*), the coefficient (a) of *ER* is −1.046, which is significant at the 10% level. This indicates that environmental regulations exert a notable adverse influence on the mediating variable of independent directors.

Furthermore, when further analyzing the impact of independent directors (*Indep*) on the environmental investments (*EnvirExpend*) of listed companies, the empirical results in Column (3) show that the coefficient b of the proportion of independent directors (*Indep*) is –0.358 and significantly at the 5% level. This indicates that independent directors significantly negatively impact on the green environmental investments of listed companies. This conclusion is inconsistent with the findings of Cosma et al. (2021), who argue that independent directors significantly promote a company’s green environmental investments. They believe that independent directors can help companies establish an environmental reputation by encouraging enterprises to participate in government environmental protection investments so as to achieve long-term development for the company. The reason why independent directors appear to inhibit the green environmental investment of listed companies in this paper is mainly because independent directors represent the interests of small and medium-sized shareholders and supervise the decision-making process of the company from an objective and independent perspective (Jensen, 1993). When faced with substantial green environmental investments, independent directors may be more cautious in assessing the risks and returns of projects. If they perceive that the short-term returns of environmental investment are not significant or present significant risks, they might tend to suppress such investment (Dwekat et al., 2020).

Simultaneously, the coefficient c’ for environmental regulation (*ER*) is-10.718, showing a significant impact at the 1% level. This suggests that environmental regulation has a direct effect of −10.718 on the environmental investment of listed companies. It further shows that the significant relationship between environmental regulation and environmental investment of listed companies has remained the same after adding independent directors (*Indep*). However, the coefficient of *ER* has changed from c = −10.344 to c’ = −10.718, and the coefficient b between the proportion of independent directors and environmental investment of listed companies is-0.358 and significantly at the 5% level. This indicates that independent directors (*Indep*) partially mediate the relationship between environmental regulation (*ER*) and environmental investment (*EnvirExpend*) of listed companies.

At this point, the mediation effect is 0.374 (ab = −1.046*-0.358 = 0.374), accounting for −3.60% of the total effect. This shows that having independent directors on the board leads to a decrease of 0.374 in the impact of environmental regulations on listed companies’ environmental investments. This suggests that the presence of independent directors in corporate governance helps oversee companies’ decisions regarding environmental investments.

In particular, the presence of independent directors can mitigate the potential constraints imposed by environmental regulations on companies’ investments in environmental protection and restricting managers’ opportunistic behaviors, thus playing an essential role in balancing short-term economic goals and long-term sustainable development goals (including green environmental investments) (Li et al., 2014).

## 5 Heterogeneity analysis and robustness test

### 5.1 Heterogeneity analysis

#### 5.1.1 Regression results of the sample divided by industry type

To test whether the impact of environmental investment on listed companies’ productivity differs across industries, this paper classifies listed companies into heavily and non-heavily polluting industries. The categorization is determined by the “*Industry Classification Management Directory for Environmental Inspection of Listed Companies*” created in 2008 and the “*Guidelines for Environmental Information Disclosure of Listed Companies*” established in 2010 by the Ministry of Ecology and Environment in China.

The research findings (see Table 7) indicate that in heavily polluting industries, the regression coefficient of environmental regulation (*ER*) is significantly negative at the 1% level. This shows that environmental regulations have a notable adverse effect on the environmental investments made by listed companies in polluting industries. In contrast, within non-heavily polluting industries, the regression coefficient of environmental regulation policy (*ER*) is not significant, suggesting that environmental regulation policy does not have a substantial effect on the environmental investments made by listed companies in non-heavily polluting industries. These results contrast with those of Wang et al. (2021), who propose that environmental regulations encourage environmental investments by companies with high energy consumption and emissions. This is mainly due to the implementation of green credit policies, which promote the optimization of resource allocation for high energy consumption and high emission enterprises, thereby improving the efficiency of their environmental investments.

**Table 7:** Results of industry heterogeneity in the impact of environmental regulations on firms’ environmental investment.

	(1)	(2)
Variable	Polluting industry	Non-polluting industry
*ER*	−15.228***	2.300
	(−3.34)	(0.51)
*S0E*	0.067*	−0.043
	(1.90)	(−0.72)
*FirmAge*	−0.015	−0.392
	(−0.07)	(−1.35)
*Size*	−0.185***	−0.143***
	(−7.50)	(−3.63)
*ROE*	0.026	−0.061
	(0.50)	(−0.66)
*Top*	−0.061	−0.420*
	(−0.50)	(−1.67)
*Lev*	0.036	−0.191
	(0.41)	(−1.29)
*Growth*	0.010	0.011
	(0.50)	(0.71)
*Cashflow*	−0.145	0.429
	(−1.21)	(1.08)
*Constant*	11.891	−9.166
	(0.43)	(−0.29)
*R-squared*	0.233	0.186
*Company FE*	YES	YES
*Year FE*	YES	YES
*Number of listed companies*	175	71
*Empirical p-values*	0.012	

The values of *t* are in parentheses, ***, **, and * denote the significance at the 1, 5, and 10% levels, respectively. To control for possible heteroskedasticity and autocorrelation, heteroskedasticity robust standard errors were used for the model.

Listed companies in polluting industries face challenges in making environmental investments due to the restrictions imposed by environmental regulations. This is because enterprises in these industries generate a large amount of pollutants during the production process. Governments impose relatively higher environmental information disclosure and regulation requirements on these enterprises. Companies must invest more funds in purchasing environmental protection technology and equipment to meet the requirements of environmental governance, which could potentially increase compliance costs and reduce economic benefits. When companies face significant cost pressures, they prioritize short-term financial benefits. Furthermore, the return on investment in environmental protection is long-term and uncertain, which eventually leads to companies being reluctant to increase their environmental investments. Thus, the stricter the government’s environmental regulation policies are, the stronger the inhibition of listed companies’ environmental investment. In contrast, enterprises in non-polluting industries generate fewer pollutants during production and require relatively less investment in environmental protection. Therefore, environmental regulation policies have a less direct impact on environmental investments.

Further analysis of the inter-group coefficient difference test for environmental regulation (*ER*) reveals that the empirical *p*-value rejects the null hypothesis at a significance level of 5%. This indicates that the impact of environmental regulation (*ER*) on the environmental investments of listed companies shows significant differences between companies in polluting sectors and those in non-polluting sectors.

#### 5.1.2 Regression results of the samples by company asset size

To analyze the heterogeneous impact of environmental regulation on green environmental investments of listed companies at different asset size levels, this study divides the sample into two groups based on the mean value of total assets in listed companies: small asset size group and large asset size group. The regression results are shown in Columns (1) and (2) of Table 8.

**Table 8:** Regression results of the impact of environmental regulation on the size heterogeneity of firms’ environmental investment.

	(1)	(2)
Variable	Small asset size group	Large asset size group
*ER*	−33.697***	−7.503*
	(−3.20)	(−1.83)
*S0E*	−0.078	0.060**
	(−0.86)	(2.36)
*FirmAge*	0.241	−0.162
	(0.52)	(−1.03)
*Size*	−0.161***	−0.175***
	(−4.53)	(−7.76)
*ROE*	0.000	0.019
	(0.00)	(0.37)
*Top*	−0.373	−0.155
	(−1.52)	(−1.28)
*Lev*	−0.090	−0.083
	(−0.56)	(−1.02)
*Growth*	0.022	0.011
	(0.92)	(0.63)
*Cashflow*	−0.539	0.102
	(−1.57)	(0.67)
*Constant*	47.807	5.066
	(0.89)	(0.26)
*R-squared*	0.190	0.225
*Company FE*	YES	YES
*Year FE*	YES	YES
*Observations*	123	123
*Empirical p-values*	0.237	

The values of t are in parentheses, ***, **, and * denote the significance at the 1, 5, and 10% levels, respectively. To control for possible heteroskedasticity and autocorrelation, heteroskedasticity robust standard errors were used for the model.

From Table 8, it can be seen that in both the small and large asset size groups, environmental regulation (*ER*) has a significantly negative impact on the green environmental investment of listed companies at the 1 and 10% level, respectively. The regression coefficients for *ER* are −33.697 and −7.503, respectively. This finding suggests that small-sized companies are more significantly impacted by environmental regulation policies in terms of their green environmental investment compared to large-sized companies. This finding contrasted with the study by Hoogendoorn et al. (2015), who pointed out that the company’s size had the most significant impact on enterprises’ participation in green production processes and other environmental investments. Furthermore, compared to large enterprises, small and medium-sized enterprises have a relatively lower willingness to invest in environmental protection. Strict environmental regulations only promote the active involvement of small and medium-sized enterprises in green products and services, but the impact on green production processes is not evident.

The reason why environmental regulations hinder environmental investment in small listed companies more than in large listed companies in this paper is mainly because the tightening of environmental regulations may result in higher production costs for smaller companies. This increase in costs may trigger a rise in product prices and a decline in market competitiveness, ultimately causing a negative impact on the profitability of enterprises. Consequently, this may affect the willingness of enterprise managers to invest in green environmental protection initiatives of listed companies (Garriga and Mele, 2004). This result aligns with the principal-agent theory proposed by Friedman (1970). As agents of listed companies, company managers often perceive green environmental investment activities as a way to fulfill personal goals rather than considering shareholders’ perspectives when deciding on corporate environmental investment activities. The inconsistency of interests may lead managers to prioritize the short-term financial goals of the company. Therefore, managers have little enthusiasm for green investments, which have uncertain returns and long-term characteristics.

In contrast, due to the economies of scale effect, large-sized listed companies may find that the long-term benefits of environmental investments (such as reduced fines, enhanced brand image, etc.) can offset the increase in short-term additional environmental costs. Therefore, the willingness to invest in environmental protection is higher among large-sized listed companies than smaller-sized ones. As a result, environmental regulations have a more significant impact on restricting the environmental investments of small listed companies compared to large-sized ones.

In addition, the inter-group coefficient difference test for environmental regulation (*ER*) between the small and large asset size groups reveals an empirical *p*-value of 0.237, which leads to the acceptance of the null hypothesis at the 10% level. This indicates that the impact of environmental regulation on the environmental investment of listed companies does not show significant differences among different asset size groups.

#### 5.1.3 Regression results of the samples by region

To further explore how environmental regulations affect the environmental investments of listed companies in various regions, these companies are categorized into eastern, central, and western regions based on their registration locations.

The research findings (see Table 9) indicate that environmental regulation policies significantly inhibit the environmental investments of listed companies in the Western region. However, this impact is not significant for companies in the Eastern and Central regions. This conclusion contradicts the findings of Hu et al. (2023), who indicated that stricter environmental regulations significantly reduce environmental investments by listed companies in the Eastern region but have a less significant effect on companies in the Central and Western regions. The reason why environmental regulation policies hinder the increase in environmental investments of listed companies in the Western region is partly because, compared to the Eastern and Central regions, the Western region has a relatively lower level of economic development, a lower degree of marketization, and the size and technological level of listed companies are relatively weaker (Lei et al., 2017). Therefore, when facing the strict restrictions and requirements of environmental regulation policies, companies in the Western region may be unable to bear the additional environmental costs due to constraints in funding, technology, and management, thereby hindering their environmental investments.

**Table 9:** Regression results of regional heterogeneity in the impact of environmental regulations on firms’ environmental investment.

	(1)	(2)	(3)
Variable	Eastern region	Central region	Western region
*ER*	−6.404	−13.259	−21.202***
	(−1.63)	(−1.08)	(−2.68)
*S0E*	0.038	−0.120	−0.016
	(1.14)	(−0.66)	(−0.18)
*FirmAge*	−0.040	−0.669	0.090
	(−0.16)	(−1.56)	(0.30)
*Size*	−0.165***	−0.191***	−0.162***
	(−6.02)	(−3.87)	(−4.05)
*ROE*	−0.010	−0.137	0.069
	(−0.18)	(−0.87)	(0.87)
*Top*	−0.203	0.031	−0.047
	(−1.22)	(0.10)	(−0.35)
*Lev*	−0.059	−0.136	−0.037
	(−0.52)	(−0.70)	(−0.38)
*Growth*	0.008	0.038	−0.003
	(0.38)	(0.86)	(−0.18)
*Cashflow*	−0.052	0.237	0.019
	(−0.35)	(0.54)	(0.10)
*Constant*	15.525	−63.730	48.509
	(0.52)	(−1.41)	(1.14)
*R-squared*	0.205	0.239	0.210
*Company FE*	YES	YES	YES
*Year FE*	YES	YES	YES
*Number of listed companies*	129	53	64
*Empirical p-values*			
*East versus Central*	0.129		
*East versus West*	0.107		
*Central versus West*	0.491		

The values of t are in parentheses, ***, **, and * denote the significance at the 1, 5, and 10% levels, respectively. To control for possible heteroskedasticity and autocorrelation, heteroskedasticity robust standard errors were used for the model.

On the other hand, the industrial structure of the Western region may be relatively traditional and homogeneous, with a high dependency on the development and utilization of heavy-polluting industries, such as energy resources. Increasing the intensity of environmental regulation policies may cause these enterprises to face higher environmental standards and cost pressures. Coupled with the relatively lenient enforcement of environmental policies, this leads to a weak willingness to invest in environmental protection among listed companies in the Western region (Zhao and Sun, 2016). Moreover, compared to companies in the Eastern and Central regions, the capital market and financing environment in the Western region are relatively immature, with limited financing channels for enterprises and higher financing costs. This might restrict the investment capacity and willingness of Western enterprises in the field of environmental protection.

In addition, the test for differences in environmental regulation (*ER*) among listed companies in the Eastern, Central, and Western regions revealed that the empirical *p*-values are 0.129 for the comparison between the Eastern and Central regions, 0.107 for the Eastern and Western regions, and 0.491 for the Central and Western regions, respectively. These findings show that the influence of environmental regulation on the environmental investments of listed companies does not vary significantly across the Eastern, Central, and Western regions of China.

### 5.2 Robustness test

#### 5.2.1 Robustness test of the baseline regression

In the previous analysis of the baseline regression that evaluated the influence of environmental regulation on the environmental investment of listed companies, standard errors was conducted by using clustered robust standard errors at the company level. For panel data of listed companies, disturbance terms within the same industry may be correlated. Therefore, it is necessary to perform robustness tests on the standard error clustering of benchmark regression models at the industry level.

After empirical testing (see Table 10), we find that the coefficient of *ER* remains statistically at the 1% level, with no change in the coefficient value. There was a small adjustment in the t-value for environmental regulation (*ER*), but the significance of environmental regulation (*ER*) remained unchanged. Therefore, this proves that the baseline regression model is robust.

**Table 10:** Robustness test results of the baseline regression.

Variable	(1)	(2)
*ER*	−10.311***	−10.344***
	(−3.12)	(−3.00)
*S0E*	0.028	0.028
	(0.78)	(0.78)
*FirmAge*	−0.149***	−0.124
	(−2.87)	(−0.67)
*Size*	−0.169***	−0.169***
	(−7.95)	(−7.93)
*ROE*	−0.009	−0.010
	(−0.23)	(−0.23)
*Top*	−0.134	−0.133
	(−1.14)	(−1.14)
*Lev*	−0.053	−0.056
	(−0.78)	(−0.86)
*Growth*	0.007	0.007
	(0.84)	(0.84)
*Cashflow*	0.033	0.034
	(0.24)	(0.25)
*Constant*	4.463***	7.720
	(11.13)	(0.32)
*R-squared*	0.206	0.206
*Number of listed companies*	246	246
*Company FE*	YES	YES
*Year FE*	NO	YES

*t*-values are in parentheses, ***, **, and * denote the significance at the 1, 5, and 10% levels, respectively. To prevent possible heteroscedasticity and autocorrelation problems, the industry-level clustered robust standard errors was used to estimate the model. In order to further verify the robustness of the benchmark regression, this paper also replaced the explained variable of EnvExpend with the logarithm of the enterprise’s green environment investment, and controlled the industry-level clustering robust standard error. We found that the coefficients of the benchmark regression did not change significantly, and the result was not presented here due to space limitations.

#### 5.2.2 Robustness test of the mediation effect analysis

In the previous section, we utilized the stepwise test method to investigate how independent directors impact the relationship between environmental regulation and environmental investment in listed companies. In order to analyze the robustness of the mediation effect, this study initially replaces *EnvirExpend* (the proportion of the current period’s environmental expenditure to the total assets of the company) with *lnEnvirExpend* (the logarithm of the current period’s environmental expenditure amount). Then, a robustness test of the mediation effect is performed by using the Sobel test method as proposed by Sobel (1982).

The research results show (see Table 11) that the p-value for the mediation effect is 0.072, which is less than 0.10, indicating the existence of the mediation effect, and the mediation effect accounts for −11.70% of the total effect, suggesting the presence of a partial mediation effect. Furthermore, compared to the mediation effect results in the previous section (see Table 6), we observe that, apart from the overall effect that was found to be insignificant, all other variables have shown significance in the test. This proves that the mediation effect is robust and remained unchanged. Therefore, this proves that the baseline regression model is robust.

**Table 11:** Robustness test results of the mediation effect regression.

Variable	Model (1)	Model (2)	Model (3)
	lnEnvirExpend	Indep	lnEnvirExpend
*ER*	−22.028	−1.046**	−24.603*
	(−1.50)	(−2.24)	(−1.68)
*Indep*			−2.462***
			(−3.02)
*S0E*	0.015	0.008***	0.034
	(0.14)	(3.08)	(0.32)
*FirmAge*	−0.007	0.020	0.042
	(−0.01)	(1.32)	(0.08)
*Size*	0.050	−0.000	0.050
	(1.20)	(−0.01)	(1.20)
*ROE*	0.318*	−0.013*	0.287
	(1.76)	(−1.92)	(1.59)
*Top*	−0.541	−0.006	−0.557
	(−1.25)	(−0.60)	(−1.28)
*Lev*	0.348	−0.007	0.330
	(1.46)	(−1.06)	(1.38)
*Growth*	0.007	−0.001	−0.062
	(−1.24)	(−0.58)	(−1.27)
*Cashflow*	−0.077	0.018	−0.033
	(−0.18)	(1.49)	(−0.08)
*Constant*	63.260	2.252	68.805
	(0.98)	(1.26)	(1.07)
*R-squared*	0.647	0.600	0.650
*Number of listed companies*	246	246	246
*Company FE*	YES	YES	YES
*Year FE*	YES	YES	YES

The values of *t* are in parentheses, ***, **, and * denote the significance at the 1, 5, and 10% levels, respectively. To control for possible heteroskedasticity and autocorrelation, firm-level clustering robust standard errors were used for the model. The method of sobel test was used to analyze the mediation effect analysis. In order to further verify the robustness of the mediation effect, this paper also conducted a test using the bootstrap method, and the results showed that the mediation effect also exists, which is not presented here due to the limitation of space.

## 6 Further discussion: marginal effect analysis of environmental regulation on the environmental investment of listed companies

In order to analyze the impact of environmental regulation policies on the marginal effect of enterprises’ environmental investment, this paper used the quantile regression model proposed by Koenker and Bassett Jr (1978) to analyze the impact of environmental regulation policies on 25, 50 and 75% quantiles of enterprises’ green environmental investment. The research results show in Table 12.

**Table 12:** Quartile regression results of the impact of environmental regulation on firms’ environmental investment.

	(1)	(3)	(4)
Variable	25% quantile	50% quantile	75% quantile
*ER*	−0.598***	−1.172***	−13.406***
	(−6.87)	(−3.34)	(−88.96)
*S0E*	−0.010***	−0.016***	−0.034***
	(−9.33)	(−18.69)	(−100.76)
*FirmAge*	0.000	0.007***	−0.012***
	(0.07)	(5.13)	(−13.31)
*Size*	−0.011***	−0.032***	−0.072***
	(−31.52)	(−36.28)	(−210.01)
*ROE*	−0.002	0.026***	0.062***
	(−0.62)	(7.91)	(26.98)
*Top*	0.021***	0.079***	0.176***
	(7.73)	(14.49)	(102.15)
*Lev*	0.009***	0.029***	0.184***
	(3.10)	(14.41)	(123.34)
*Growth*	−0.004***	0.003***	0.022***
	(−11.90)	(4.18)	(21.88)
*Cashflow*	0.062***	0.109***	0.270***
	(5.21)	(17.54)	(62.80)
*Number of listed companies*	246	246	246

The values of *t* are in parentheses, ***, **, and * denote the significance at the 1, 5, and 10% levels, respectively. To control for possible heteroskedasticity and autocorrelation, firm-level clustering robust standard errors were used for the model. The seed value is set to 10,101, and considering the possibility of endogeneity of ER, we use instrumental variable method to deal with endogenous variables and Markov chain Monte Carlo method (MCMC) to estimate the results.

The results indicate that the regression coefficients for *ER* at three different quantiles are −0.598, −1.172, and −13.406, respectively. These coefficients exhibit statistical significance at the 1% level. The analysis reveals that the regression coefficients for *ER* at each quantile are significantly negative, indicating that stringent environmental regulation policies significantly reduce corporate environmental investment. These results suggest that as the quantiles rise, there is a noticeable upward trend in the absolute values of the quantile regression coefficients for environmental regulation. This trend indicates that the impact of stricter environmental regulations on the environmental investments of listed companies becomes more pronounced as the quantiles increase.

## 7 Conclusion and policy recommendations

As environmental issues gain increasing prominence in China, the Chinese government has enacted stringent environmental regulations and standards to encourage enterprises to increase their investments in environmental protection. However, relying solely on government regulation is insufficient. Listed companies also need to recognize the importance of environmental investment to achieve coordinated economic and environmental development. Simultaneously, supervising independent directors is crucial for encouraging listed companies to increase their environmental investments. Independent directors can ensure corporate compliance and sustainability in environmental protection by overseeing corporate environmental policies and performance.

In light of this, the paper focuses on the actual situation of environmental protection investment by listed companies. We investigate how environmental regulations have influenced the environmental protection investments made by 246 listed companies in China from 2010 to 2019. Then, we analyze the mediating role of independent directors in board governance in shaping the connection between environmental regulations and environmental investments of these firms. In addition, we explore the heterogeneous impact of environmental regulations on environmental protection investments by listed companies across different industries, asset sizes, and regions. The main conclusions of the study are as follows:

First, within the sample period, environmental regulations significantly negatively impact environmental investment of listed companies. This indicates that stricter environmental regulations greatly impede the growth of corporate environmental investments. Moreover, the stronger the government’s environmental regulation, the more pronounced the inhibition on listed companies’ environmental investments will be.

Second, within the sample period, the presence of independent directors diminishes the constraining impact of environmental regulations on environmental investments made by listed companies. This conclusion suggests that independent directors mitigate the adverse effects of environmental regulations on environmental investments by listed companies. It also highlights the importance of having independent directors on boards to evaluate government environmental policies and oversee corporate decisions related to environmental protection investments.

Third, within the sample period, heterogeneity analysis results indicate that environmental regulation policies negatively impact on the environmental investment of listed companies in pollution-intensive industries and those located in the western regions of China. Furthermore, the inhibitory effect of environmental regulation policies on the environmental investment of small-sized companies is significantly higher than that of large-sized companies.

Finally, within the sample period, the panel quantile regression results show that the strengthening of environmental regulatory policies has an increasingly significant inhibitory effect on green environmental investment by listed companies.

Based on the above conclusions, this paper presents the following policy suggestions. Firstly, it is imperative for the government to thoroughly assess the implications of environmental regulation policies on the environmental investments of listed companies. It is important to avoid overly strict or lenient policies that inappropriately inhibit corporate environmental investment, ensuring the rationality and feasibility of the policies. Moreover, the government should promote and guide enterprises to correctly understand the intentions of environmental policies through publicity and guidance. This would reduce resistance and misunderstanding regarding the policies and enhance the willingness and initiative of enterprises to invest in environmental protection.

Secondly, policymakers should encourage and guide listed companies to further improve the independent director system, enhancing independent directors’ independence and supervisory capabilities. The decision-making process of corporate green investment is influenced by a variety of psychological factors, such as risk appetite and expected returns. The monitoring role of independent directors stems from the enhancement of the psychological security of enterprises. Therefore, from the perspective of organizational psychological security, the improvement of professional competence of independent directors can help enterprises to cope with the pressure of environmental regulation and improve the level of green investment. Thus, the government could create training programs and offer incentives to independent directors, motivating them to effectively utilize their expertise in guiding and supervision of corporate environmental investments. By enhancing independent directors’ environmental awareness and professional capabilities, they can be more equipped to participate effectively in the environmental investment decisions and supervision of enterprises.

Finally, the government should formulate differentiated environmental regulation policies for enterprises in different industries, sizes, and regions. For companies operating in pollution-intensive industries and western regions, the government could adopt stricter environmental standards, emission restrictions, environmental monitoring, and administrative penalties to limit pollution emissions, compelling enterprises to increase their investments in environmental protection and technological innovation. For small-size listed companies, the government should establish more flexible and moderate environmental regulation policies and create specialized financial mechanisms to provide these enterprises with low-interest loans or venture capital. This would reduce the costs and risks associated with their environmental investments and alleviate the pressure of such investments.

Looking to the future, there are large differences in environmental subsidies for technological innovation among Chinese provinces, which have an important impact on the environmental investment decision-making behavior of listed firms. In the future, a policy assessment can be carried out around the environmental subsidy policy on the environmental investment of listed firms. On the other hand, the psychological characteristics of enterprise leaders (such as environmental responsibility, innovative spirit, etc.) may affect the decision-making of enterprises’ green investment. In the future, we can explore the relationship between the psychological characteristics of leaders and environmental investment, which will help to understand how the psychological characteristics of enterprise leaders affect the environmental investment decisions of listed enterprises.

## Data Availability

The raw data supporting the conclusions of this article will be made available by the authors, without undue reservation.
